# Perfluorodecalins and Hexenol as Inducers of Secondary Metabolism in *Taxus media* and *Vitis vinifera* Cell Cultures

**DOI:** 10.3389/fpls.2018.00335

**Published:** 2018-03-16

**Authors:** Heriberto R. Vidal-Limon, Lorena Almagro, Elisabeth Moyano, Javier Palazon, Maria A. Pedreño, Rosa M. Cusido

**Affiliations:** ^1^Secció de Fisiologia Vegetal, Facultat de Farmacia, Universitat de Barcelona, Barcelona, Spain; ^2^Department of Plant Biology, Faculty of Biology, University of Murcia, Murcia, Spain; ^3^Departament de Ciències Experimentals i de la Salut, Universitat Pompeu Fabra, Barcelona, Spain

**Keywords:** cell cultures, gene expression, hexenol, perfluorodecalins, resveratrol, taxanes, *Taxus media*, Vitis vinifera

## Abstract

Plant cell cultures constitute a potentially efficient and sustainable tool for the production of high added-value bioactive compounds. However, due to the inherent restrictions in the expression of secondary metabolism, to date the yields obtained have generally been low. Plant cell culture elicitation can boost production, sometimes leading to dramatic improvements in yield, as well as providing insight into the target biosynthetic pathways and the regulation of the genes involved. Among the secondary compounds successfully being produced in biotechnological platforms are taxanes and *trans*-resveratrol (*t*-R). In the current study, perfluorodecalins (PFDs) and hexenol (Hex) were tested for the first time with *Taxus media* and *Vitis vinifera* cell cultures to explore their effect on plant cell growth and secondary metabolite production, either alone or combined with other elicitors already established as highly effective, such as methyl jasmonate (MeJa), coronatine (Coro) or randomly methylated β-cyclodextrins (β-CDs). The total taxane content at the peak of production in *T. media* cell cultures treated with PFDs together with Coro plus β-CDs was 3.3-fold higher than in the control, whereas the *t*-R production in MeJa and β-CD-treated *V. vinifera* cell cultures increased 552.6-fold compared to the extremely low-yielding control. Hex was ineffective as an elicitor *in V. vinifera* cell cultures, and in *T. media* cell suspensions it blocked the taxol production but induced a clear enhancement of baccatin III. Regarding biosynthetic gene expression, a strong positive relationship was observed between the transcript level of targeted genes and taxol production in the *T. media* cell cultures, but not with *t*-R production in the elicited *V. vinifera* cell cultures.

## Introduction

One of the most successful examples of biotechnological production of high added-value compounds is the use of *Taxus* spp. cell cultures to produce the well-known anti-cancer compound taxol as well as other taxanes used as semi-synthetic precursors of taxol and its analogs. At present, several companies are producing these compounds at an industrial level, including Phyton, Cell Therapeutics, Abraxis, and Corean Samyang Genex (Exposito et al., 2009; Malik et al., 2011). Another bioactive compound of great interest for the chemical-pharmaceutical industry is *trans*-resveratrol (*t*-R), a polyphenol belonging to the stilbene family, which has been the subject of numerous studies in medicine and plant physiology. Sources of *t*-R include extraction from plant raw material and chemical synthesis, but its biotechnological production has the advantage of combining cost-effectiveness with bio-sustainability. *t*-R is able to act as a phytoalexin in response to plant stress (Langcake and Pryce, 1976), and has cardio-protective, anticancer, neuroprotective and antioxidant properties (Adrian and Jeandet, 2006).

Bioproduction processes usually need to be improved by the addition of elicitors (Ramirez-Estrada et al., 2016b). Many studies have shown the effectiveness of supplementing *Taxus* spp. and *Vitis vinifera* cell cultures with either biotic or abiotic elicitors to increase the accumulation of taxol and related taxanes (Vongpaseuth and Roberts, 2007; Exposito et al., 2009; Onrubia et al., 2013a; Ramirez-Estrada et al., 2016b), or *t*-R (Belchí-Navarro et al., 2012; Almagro et al., 2014). We found that the addition of methyl jasmonate (MeJa) to *Taxus* spp. cell cultures at the production stage (Cusido et al., 2002) is an efficient method for enhancing taxane yield. In the same *T. media* cell cultures, the joint action of MeJa and β-CDs led to a dramatic increase in taxol production (Sabater-Jara et al., 2014). Randomly methylated β-cyclodextrins (β-CDs) are cyclic oligosaccharides formed by 7 β-D-glucopyranose units produced from the enzymatic degradation of starch by the bacterial cyclodextrin glycosyltransferase (Szejtli, 1997; Qi et al., 2007). However, we obtained the maximum levels of taxol and related taxanes using coronatine (Coro, 1 μM) together with β-CDs (50 μM). Coro, which is a phytotoxin produced by different pathovars of *Pseudomonas syringae* (Bender et al., 1999), is a natural analog of the active form of jasmonate, JA-Ile. In *T. media* cell cultures (Onrubia et al., 2013b), the yield of total taxanes increased remarkably after elicitation with Coro (1 μM), while taxol and baccatin III production was significantly enhanced by the combination of β-CDs and Coro (Ramirez-Estrada et al., 2015).

Similarly, *Vitis vinifera* cell cultures treated with β-CDs and MeJa (Bru et al., 2006; Belchí-Navarro et al., 2012; Almagro et al., 2014) constitute an efficient system for the bioproduction of *t*-R. Significantly, the *t*-R levels produced by a β-CD and MeJa treatment were greater than the sum of yields achieved by the elicitors applied individually, indicating a synergistic effect (Almagro et al., 2014). Consequently, at present, the most efficient *t*-R bioproduction platform is *V. vinifera* cell cultures elicited with CDs and MeJa, with 2% of the sucrose added to the medium being converted to *t*-R, reaching levels of around 4 g/L (for a review, see Donnez et al., 2009). The use of β-CDs allows a direct extraction of the target compound from the culture medium without biomass destruction (Belchí-Navarro et al., 2012). The extracellular levels of *t*-R obtained in *V. vinifera* cell cultures elicited with β-CDs and MeJa were at least 10-fold higher than those obtained using other methodologies (Beekwilder et al., 2006; Watts et al., 2006; Kiselev et al., 2007).

In all the aforementioned experiments, elicitation had a strong effect on gene expression: in *Taxus* spp. cultures, those involved in the biosynthetic pathway of taxol and related taxanes, (Cusido et al., 2014), and in the case of *V. vinifera*, genes upstream of the *t*-R biosynthetic pathway, as well as others involved in phenylpropanoid formation (Almagro et al., 2014).

Although the numerous elicitation studies on improving production of the target secondary metabolites have achieved some progress, yields remain low. Therefore, to enhance the productivity of *Taxus* spp. and *V. vinifera* cell cultures, it is necessary to find new elicitors and understand their action mechanisms. In this scenario, perfluorodecalin (C_10_F_18_, PFD), a fluorocarbon in which all the hydrogen atoms are replaced by fluorine atoms, is a promising candidate. This dense liquid can dissolve large volumes of non-polar gasses such as O_2_ (35–44 μMol/L), and when added to a liquid medium, it facilitates the formation of a second phase below the aqueous phase (Lowe, 2002). The addition of air-saturated PFD to tobacco cell cultures increased the cell biomass more than three-fold compared to PFD-free control cell cultures (Pilarek and Szewczyk, 2008). Although pure oxygen dissolved in PFD has a negative effect on tobacco cell biomass, this varies according to cell sensitivity to high oxygen concentrations (Pilarek and Szewczyk, 2008). In *Taxus* hairy root cultures, the addition of either aerated or degassed PFD to the culture medium increased the taxol production, especially when accompanied by 100 μM MeJa. Since PFD is not soluble in water, the hairy root cultures supplemented with PFD presented two phases, and some of the taxanes produced were found in the PFD phase. This suggests that adding PFD to the culture medium may be an effective strategy for *in situ* extraction of taxanes (Syklowska-Baranek et al., 2015).

Herbivorous attacks can induce defense mechanisms in plants that involve the accumulation of enzymes such as polyphenol oxidases and protease inhibitors, and the release of volatile organic compounds (Sugimoto et al., 2014). (Z)-3-hexenol (Hex) is a natural volatile organic compound produced in wounded green tissues that mediates in protective plant-plant interactions. The addition of this compound can also trigger defense reactions. Xin et al. (2016) showed that tea plants treated with Hex increased levels of jasmonic acid and ethylene, as well as a higher expression of several genes related with plant defense. A similar effect was observed in maize plants, namely an enhanced expression of genes related with induced defense mechanisms and metabolite biosynthesis (Farag et al., 2005).

Taking into account the aforementioned antecedents, the aim of this work was to study taxane and *t*-R production in elicited *T. media* and *V. vinifera* cell cultures, respectively, after supplementing optimum culture media (Cusido et al., 2002; Almagro et al., 2014) with the new elicitors, PFD (57.5% v/v), both gassed (PDFgas) and degassed (PDFdegas), or hexenol (40 μM Hex), in order to show if two different cell lines, both with a high capacity to produce bioactive compounds but with different biosynthetic pathways, are able to respond in a similar way to elicitation. Thus, PFDs and Hex were added to the culture media, with or without the elicitors reported to be the most effective for enhancing the yield of the target secondary metabolites: Coro (1 μM) and β-CDs (50 μM) in the case of *T. media*, and MeJa (100 μM) and β-CDs (50 μM) in *V. vinifera*. The capacity of the elicitors to induce the biosynthesis of taxanes and *t*-R, and increase their excretion from the producer cells to the culture medium, as well as their effects on the expression of several genes directly involved in the biosynthetic pathways of taxanes and *t-*R, were studied.

## Materials and Methods

### Plant Material and Elicitation

*Taxus media* cells were grown in a two-stage culture, as described previously (Cusido et al., 2002). After 12 days of growth in the optimum B5 medium for biomass formation (GM), the cultures were transferred to the corresponding optimum production medium (PM). The inoculum ratio was of 25 g fresh weight/100 mL of PM, supplemented with B5 vitamins, 30 g/L sucrose, 0.1 g/L myoinositol, plus an antioxidant solution (Kim et al., 2004), with the addition of 2 mg/L 2,4-dichlorophenoxyacetic acid, 0.1 mg/L benzyladenine and 0.5 mg/L gibberellic acid.

Elicitors were added at the beginning of the second culture stage, when cells were transferred to the PM. The assayed elicitors were: 50 μM of β-CDs (Wacker Chemie, Spain), in combination with 1 μM of Coro; (Sigma-Aldrich, Spain) (a combination referred to henceforth as CC); 575 ml/L PFDs (abcr GmbH, Karlsruhe Germany), both air-saturated (gassed) (PFDgas) and degassed (PFDdegas), and 40 μM cis-3-hexen-1-ol (Hex; Sigma-Aldrich, Spain). All compounds were filter-sterilized (0.22 μM sterile PES filters, Millipore, Iberica SA, United States) and added to give the final concentrations indicated. Both PFDgas and PFDdegas were prepared as indicated by Pilarek and Szewczyk (2008) and Syklowska-Baranek et al. (2015). For growth and taxane analysis, three flasks were harvested at days 0, 6, 12, 18, and 24 after each elicitor treatment. Cell growth and viability were determined as previously described (Exposito et al., 2010).

Cell cultures of *V. vinifera* cv Monastrell were established and maintained as previously described (Calderon et al., 1993; Belchí-Navarro et al., 2012). Cell cultures were transferred 12–14 days after subculture to the experimental conditions of this study. In the case of *V. vinifera*, cell growth does not require a change of the culture medium as the optimal culture conditions are the same for both the cell biomass generation and elicitation; therefore, grapevine cell cultures were grown in a control culture medium (referred to henceforth as CNT). Thus, cells of 4 g fresh weight were placed in 100 ml flasks and suspended in 20 ml of culture medium as described previously (Belchí-Navarro et al., 2012). Joint elicitation with 100 μM MeJa and 50 μM β-CD (referred to henceforth as MC) is known to be very effective in inducing *t*-R production and these elicitors were used as described previously (Belchí-Navarro et al., 2012). However, the eliciting effect of PFDs and Hex on the production of *t*-R in *V. vinifera* cell cultures has not been studied until now. These new elicitors were assayed in *V. vinifera* cell cultures as described for *T. media*. Analysis of growth and *t*-R production was performed in triplicate and flasks were harvested at 0, 1, 2, 3 and 7 days after each elicitor treatment. Cell growth and viability of *V. vinifera* cell cultures were determined as previously described (Belchí-Navarro et al., 2012).

### Extraction and Quantification of Taxanes and *t*-Resveratrol

Taxanes were extracted from cells and the culture medium as previously described (Onrubia et al., 2013b). Extraction from the PFD phase was performed following the same protocol used for extraction from the culture medium, since the PFDs used are not miscible with dichloromethane.

Taxane quantification was achieved by HPLC as described by Richheimer et al. (1992). The chromatographic analyses were performed in an HPLC Agilent 1100 series, and taxane separation was carried out in a SUPELCOSIL LC-F column 25 cm × 4.6 μM (Supelco, Bellefonte) using a mobile phase consisting of a mixture of water (A) and acetonitrile (B) and the timed gradient program: time (min)/%B: 0/25, 38/60, 40/60, 50/25 and 55/25 with a flow rate of 1 mL/min. Criteria for identification included retention time, UV spectra and co-chromatography with standards and peak homogeneity determined by a photodiode array detector when spiked with an authentic standard. Quantification was carried out from the calibration curve of each standard: 10-deacetylbaccatin III (DABIII), baccatin III (BIII), cephalomannine (CEPH), 10-deacethyltaxol (DAT) and taxol (TX) (Chromadex, LGC Standards, Barcelona, Spain).

*t*-R content in the culture media was analyzed by HPLC-DAD (Waters 600E, Waters 996) as described by Belchí-Navarro et al. (2012). In addition, *t*-R was extracted from the PFD phase by phase partitioning with ethyl acetate. Thus, the organic phase was collected and evaporated at 40°C in vacuum; the residue was dissolved in methanol and analyzed by HPLC-DAD. The intracellular *t*-R content was analysed from 50 mg of freeze-dried cells extracted overnight in 4 mL methanol at 4°C. The cell extracts were diluted with water to a final concentration of 80% (v/v) methanol. Then, 20 μL of the diluted extracts were filtered (Anopore 0.2 μM) and analyzed in an HPLC-DAD (Waters 600E, Waters 996) as described by Bru et al. (2006) using a Spherisorb ODS2 C-18 column (250 × 4.6 μM, 5 μM). *t*-R was identified at 306 nm and quantified by comparison with an authentic standard of >99% purity (Sigma-Aldrich, Spain).

### Quantitative Real-Time PCR (qRT-PCR)

RNA of *T. media* cells was isolated using the “RNeasy Mini Protocol for isolation of total RNA from plant cells and tissues and filamentous fungi” (Qiagen, Germany). cDNA and qRT-PCR was performed as described previously (Ramirez-Estrada et al., 2016b). Gene specific primers were designed with Primer3 software version 0.4.0 (**Supplementary Table S1**) and the amplification efficiency of each primer pair was determined empirically by 10-fold serial dilutions of cDNA and calculated as described by Qiagen. Only those primer pairs with an efficiency of over 0.8 were used (**Supplementary Table S1**). Expression levels were normalized to those of the *T. baccata tbc41* gene (Onrubia et al., 2013b). Relative expression values of the different genes were analyzed at 0, 4, 8, 12, 24, and 48 h after elicitation treatments. In all cases, each qPCR was performed with at least three independent samples.

Total RNA of *V. vinifera* cells was extracted from frozen cells (0.5 g FW) using the TRIZOL reagent (Invitrogen) as described by Almagro et al. (2015). First strand cDNA was synthesized from 0.2 mg of total RNA using the iScriptTM Select cDNA Synthesis Kit (Bio-Rad) with the Oligo (Dt) primers mix. The qRT-PCR procedures were performed using the primers designed by Lijavetzky et al. (2008) (**Supplementary Table S1**). qRT-PCR was performed as described previously (Almagro et al., 2015). The cDNA samples were analyzed with an iCycler (Bio-Rad) apparatus using SYBR Green PCR Core Reagents (Life Technologies) and the results were analyzed with the manufacturer’s software (iCycler Optical System Software, v. 3.0.6; BioRad). For each gene, the expression values were normalized with respect to the grapevine EFα gene used as a reference control, as described by Lijavetzky et al. (2008) and Almagro et al. (2014). Relative expression values of the different genes were analyzed at 0, 4, 8, 12, 24, and 48 h after elicitation treatments. In all cases, each PCR was performed with at least three independent samples.

### Statistics

The statistical analysis was performed with Microsoft Excel software. All data are the average of three measurements + SE. A multifactorial ANOVA analysis followed by a Tukey test were used for statistical comparisons. A *p*-value of <0.05 was assumed for significant differences.

## Results

With the aim of studying the effect of PFDs and Hex on growth and the production of taxane and *t*-R in *Taxus* and *Vitis* cell cultures, respectively, PFDgas, PFDdegas or Hex were supplied to elicited and non-elicited cell cultures. The elicitor combinations used in this study, CC for *T. media* and MC for *V. vinifera* cell cultures, have previously been established as highly effective in inducing taxane and *t-*R production, respectively, when applied to the culture medium (Almagro et al., 2014; Ramirez-Estrada et al., 2016b). Thus, *T. media* cells in an optimized production medium were treated with the following: PFDgas or PFDdegas; CC + PFDgas or PFDdegas; Hex or Hex + CC. The *V. vinifera* cells in an optimized culture medium (Belchí-Navarro et al., 2012) were treated with PFDgas or PFDdegas; MC + PFDgas or PFDdegas; Hex or Hex + MC (for quantities see Materials and Methods). All the results were compared with those obtained in the respective non-elicited control cell cultures. The *T. media* and *V. vinifera* cultures were maintained for 24 and 7 days, respectively, since the highest taxane and *t*-R yields were obtained during these periods.

### Effect of Elicitors on Growth of *Taxus media* Cell Cultures

**FIGURE 1:**
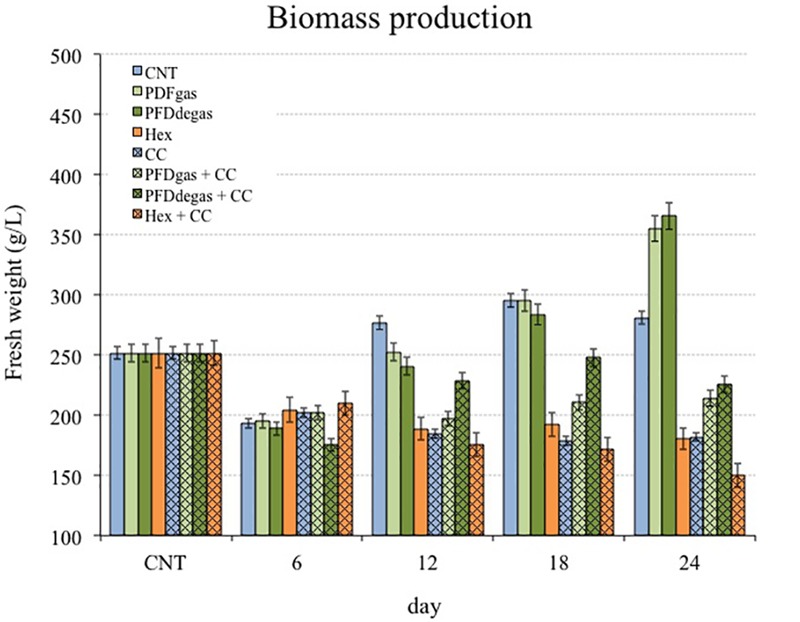
Time course of cell growth in *T. media* cell cultures measured as g FW/L. CNT, control; PDFgas, gassed perfluorodecalins; PFDdegas, degassed perfluorodecalins; PDFgas + CC, gassed perfluorodecalins plus Coro and β-CDs; PDFdegas + CC, degassed perfluorodecalins and Coro + β-CDs; Hex, Hexenol; Hex + CC, Hexenol and Coro + β-CDs. Data are the mean of three independent replicates ± SD.

The growth capacity of *T. media* cell cultures, measured as fresh weight (FW, g/L), was studied for 24 days after the cells were transferred from GM to PM, because cells grew actively in the GM (Cusido et al., 2002) but the taxane production was very low. As shown in **Figure 1**, except in the initial 6–8 day lag/adaptation phase, the growth capacity depended on the experimental conditions, despite being maintained in a medium that promotes secondary metabolism in detriment of biomass formation. Under control conditions (**Figure 1**, CNT), growth was exponential from days 6–8 to 18, and only slight thereafter. Growth was enhanced by the presence of PFDs in the PM, the biomass constantly increasing until the end of the experiment, when it was on average 1.3-fold higher than in the control (**Figure 1**, PFDgas and PFDdegas). However, when PFDs were supplemented with CC, the FW was always approximately 30% lower (*p* < 0.01) than the control (**Figure 1**, PFDgas + CC and PFDdegas + CC). The addition of Hex was significantly (*p* < 0.01) negative for growth, the final FW of the elicited cells representing only 70% of the control (**Figure 1**, Hex and Hex + CC). In contrast, in one of the few studies on the effect of Hex on plant cell cultures, Almagro et al. (2016) reported no detrimental effect on the growth of *Linum usitatissimum* cell cultures. The reduction in biomass in *T. media* cell cultures was even more pronounced when treated with Hex + CC.

### Effect of Elicitors on the Growth of *Vitis vinifera* Cell Cultures

**FIGURE 2:**
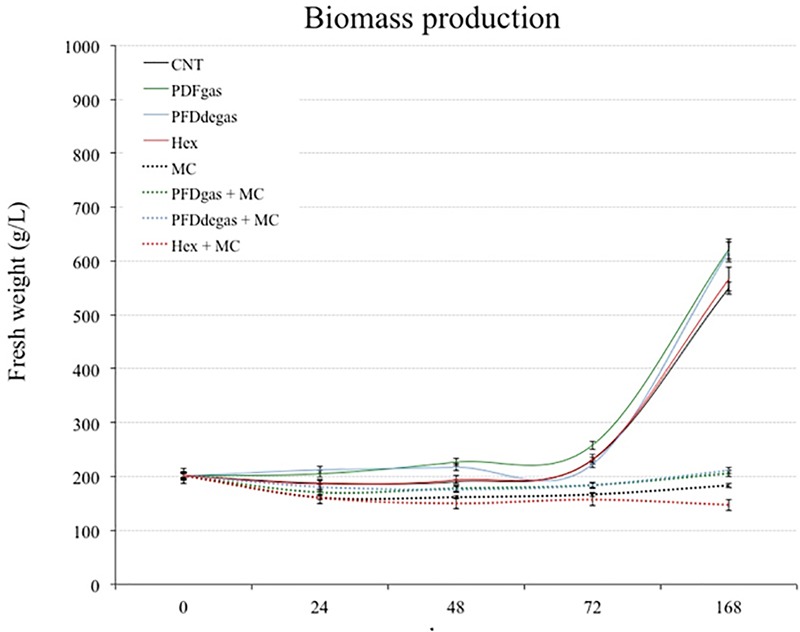
Time course of cell growth in *V. vinifera* cell cultures measured as g FW/L for 168 h. CNT, culture medium control; PDFgas, gassed perfluorodecalins; PFDdegas, degassed perfluorodecalins; PDFgas + MC, gassed perfluorodecalins plus MeJA and β-CDs; PDFdegas + MC, degassed perfluorodecalins plus MeJA and β-CDs; Hex, Hexenol; Hex + MC, Hexenol + MeJA+ β-CDs; MC, MeJA and β-CDs. Data are the mean of three independent replicates ± SD.

The biomass-forming capacity of *V. vinifera* cell cultures (measured as FW) was determined for 7 days (**Figure 2**). After an initial 3 days of only slight biomass increase, a marked exponential growth began in the control cultures and those treated with PFDs or Hex (**Figure 2**). At the end of the experiment, the increase of FW in cell cultures treated with PFDgas or degas and Hex was more than 3- and 2.8-fold higher, respectively, than in the initial conditions (200 g/L), versus 2.7-fold higher in the control cultures. In contrast, under treatment with MC, with or without PDFs or Hex, the biomass did not increase, and even decreased after treatment with Hex + MC (*p* < 0.01, in relation to the control) (**Figure 2**).

### Effect of Elicitors on Taxane Production in *Taxus media* Cell Cultures

**FIGURE 3:**
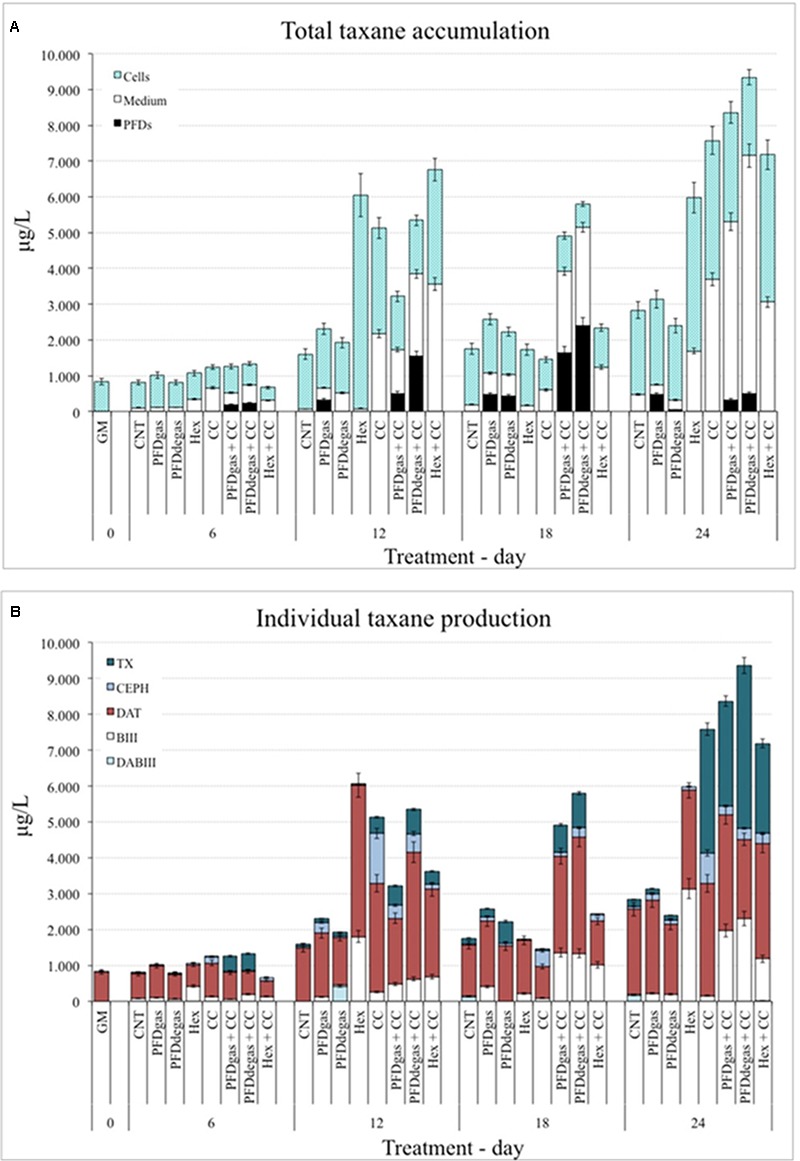
**(A)** Total taxanes accumulated inside the cells, culture medium and PFD phase at 24 days of the *T. media* cell cultures. GM, biomass production medium; CNT, control; PDFgas, gassed perfluorodecalins; PFDdegas, degassed perfluorodecalins; PDFgas + CC, gassed perfluorodecalins plus Coro and β-CDs; PDFdegas + CC, degassed perfluorodecalins and Coro + β-CDs; Hex, Hexenol; Hex + CC, Hexenol and Coro + β-CDs. Data are the mean of three independent replicates ± SD. **(B)** Individual taxane production in *T. media* cell cultures at 24 days of culture. GM: biomass production medium; CNT, control; PDFgas, gassed perfluorodecalins; PFDdegas, degassed perfluorodecalins; PDFgas + CC, gassed perfluorodecalins plus Coro and β-CDs; PDFdegas + CC, degassed perfluorodecalins and Coro + β-CDs; Hex, Hexenol; Hex + CC, Hexenol and Coro + β-CDs. Data are the mean of three independent replicates ± SD.

The total and individual taxane production was determined by HPLC in both cells and culture medium, and in the phase of PFD-supplemented cell cultures formed by this immiscible compound in an aqueous medium (**Figure 3**). The target taxanes identified and quantified were DABIII, BIII, TX, DAT and CEPH (**Figure 3B**), and the total taxane content corresponded to the sum of these five compounds (**Supplementary Table S2**).

Under control conditions, the total taxane accumulation in *T. media* cell cultures increased steadily during the 24 days of the experiment (**Figure 3A**). The highest levels of total taxanes achieved in the CC-treated cell cultures were found at days 12 and 24 after elicitation, being 3.2- and 2.7-fold higher, respectively, compared to the control (**Figure 3A**). The total taxane production was enhanced further when *T. media* cell cultures were supplemented with CC + PFDdegas (*p* < 0.01) (**Figure 3A**). Under the latter conditions, the increase was already notable at 12 days of elicitation, and the highest levels were achieved at day 24 (9.4 mg/L). The total taxane levels at days 12, 18, and 24 were on average more than threefold higher than in the control (**Figure 3A**). Elicitation with PFDgas alone enhanced the total taxane accumulation throughout the studied period, albeit only slightly (*p* > 0.05). These results indicate that the presence of PFDs in the medium enhanced the effect of CC on the taxane production.

Under Hex treatment, the total taxane production also peaked at days 12 and 24 after elicitation, levels being 3.7- and 2.1-fold higher, respectively, than in control conditions. Except at days 6 and 18, the average total taxane production was significantly higher (*p* < 0.01) (more than double) in the Hex-treated cultures than in those treated only with PFDs (**Figure 3A**). When Hex and CC were applied together, especially at days 12 and 24, the total taxane levels were lower than the sum of yields achieved separately, suggesting that these elicitors might interfere with each other’s mechanisms of action. Further studies on their receptors and signal transduction pathways are needed to understand this effect.

On the other hand, under the best production conditions, the taxanes were mainly accumulated in the culture medium rather than the cells or PFD phase (**Figure 3A**). In control conditions more than 80% of the taxanes remained inside the *T. media* cells throughout the experiment, their excretion increasing only slightly when PFDs (both gas and degas) were added (**Figure 3A**). The possible rate-limiting steps involved in taxol secretion (Collins-Pavao et al., 1996) can be reversed by elicitors, since after the addition of CC almost half of the total taxol was found in the culture medium. The same effect was induced by treatment with PFDs + CC: after 12–18 days of culture, the percentage of the total taxanes in the PFD phase increased from 25% to approximately 40%. Notably, when the production was at its highest (at day 24 in the presence of the three elicitors, PFDdegas + CC), most of the taxanes produced had accumulated in the culture medium (**Figure 3A**). When the cultures were supplemented with Hex, the cell-associated taxanes at days 6, 12, and 18 after the elicitation represented almost the totality produced, but under Hex + CC, more than 50% were released (**Figure 3A**).

Regarding individual taxane production (**Figure 3B** and **Supplementary Table S2**), the main taxane found throughout these elicitation experiments in almost all cases was DAT, which was also predominant in a *T. globosa* cell line (Ramirez-Estrada et al., 2015). In fact, in control cultures at day 24, DAT levels increased up to 2.4 mg/L, while amounts of DABIII (0.18 mg/L), BIII (0.02 mg/L), TX (0.2 mg/L) and CEPH (0.08 mg/L) were low (**Figure 3B**). Indeed, while the four latter metabolites represented at most 25% of the total taxanes produced throughout the experiments, in the control cultures, the amount of DAT was always higher than 70% (**Supplementary Table S2**). Strikingly, when CC were added to the culture medium, almost 95% of the total taxanes produced had a lateral chain (at days 12 and 24 the taxanes consisted of DAT: 60% and 40%; TX: 9% and 45%; CEPH: 27% and 11%, respectively).

Under elicitation with CC + PFD (gas or degas), the main taxane accumulated after 18 days was also DAT, although at the end of the experiments the TX levels represented 40 and 50% of the total taxanes in cell cultures treated with CC + PFDgas or PFDdegas, respectively (**Figure 3B**). Under both these treatments BIII levels increased up to approximately 25% at day 18, while CEPH and DABIII never exceeded 15% (**Supplementary Table S2**).

Under Hex treatment, the main taxane produced was also DAT, although BIII represented 30 and 52% of the total at 12 and 24 days after elicitation, respectively. It is notable that TX was not found in the Hex-treated cultures (**Figure 3B** and **Supplementary Table S2**). Considering the high levels of BIII, any biosynthetic limitation in the TX pathway would be after the step producing this TX precursor. Moreover, as DAT was the predominant taxane, the limiting step was clearly not the addition of the side chain. A possible explanation for the absence of TX is a rapid metabolization to other taxanes. Treatment with CC, with or without Hex, did not significantly (*p* > 0.05) increase the TX content at days of low taxane production (6 and 18 days of culture), but at days 12 and 24 CC induced a high TX yield, which was only slightly affected by the addition of Hex.

### Effect of Elicitors on *Trans*-Resveratrol Production in *Vitis vinifera* Cell Cultures

**FIGURE 4:**
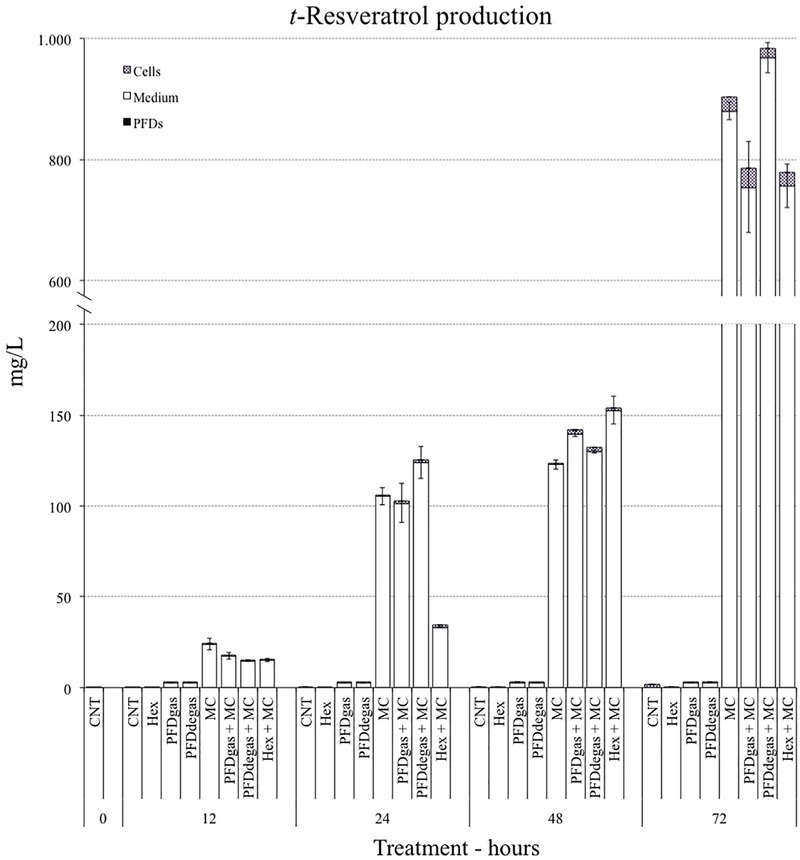
Total *trans*-resveratrol accumulated inside the cells, culture medium and PFD phase at 72 h of the *V. vinifera* cell cultures. CNT, control; PDFgas, gassed perfluorodecalins; PFDdegas, degassed perfluorodecalins; PDFgas + MC, gassed perfluorodecalins plus MeJA and β-CDs; PDFdegas + MC, degassed perfluorodecalins plus MeJA and β-CDs; Hex, Hexenol; Hex + MC, Hexenol + MeJA+ β-CDs; MC, MeJA and β-CDs. Data are the mean of three independent replicates ± SD.

The *t*-R contents of the *V. vinifera* cell cultures in all the elicitation conditions were studied for a period of 7 days. As can be observed in **Figure 4**, only treatments including MC increased the production of *t-*R very significantly (*p* < 0.01). After 24 or 48 h of elicitation, the *t-*R levels in all MC treatments, with or without PFDs or Hex, were low, with no significant differences (*p* > 0.05) between them. After 72 h, the maximum *t-*R levels were obtained with MC and PDFdegas (983 mg/L), followed by MC (903 mg/L). The effect of the MC treatment on *t*-R production was similar when co-administered with PFDgas or Hex (785 mg/L and 779 mg/L, respectively). These results clearly indicate that the high levels of *t-*R were associated with the presence of MC in the culture medium (**Figure 4**). The addition of PFDdegas to the MC treatment enhanced the *t*-R production by 8% (*p* < 0.05), while PFDgas and Hex reduced it by 14%.

Moreover, in the elicited *V. vinifera* cell cultures after 72 h, *t*-R was mainly detected in the culture medium, with the following proportions found in the cells in relation to the total production after the different treatments: MC (2.5%), PFDgas + MC (4%), PFDdegas + MC (1.5%) and Hex + MC (3%) (**Figure 4**).

### Effect of Elicitors on the Transcriptomic Profile of Elicited *Taxus media* Cell Cultures

**FIGURE 5:**
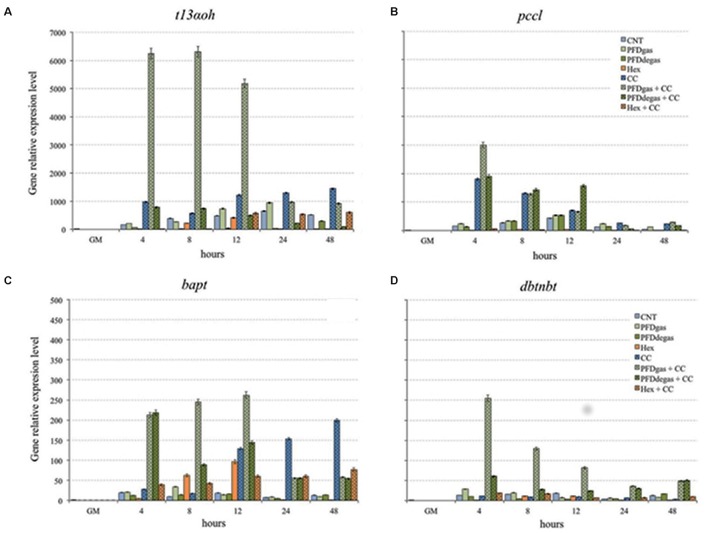
Relative expression level of *t13αoh* gene **(A)**, *pccl* gene **(B)**, *bapt* gene **(C)**, and *dbtnbt* gene **(D)** in *T. media* cell cultures elicited at 48 h of culture. GM, growth medium; CNT, control; PDFgas, gassed perfluorodecalins; PFDdegas, degassed perfluorodecalins; PDFgas + CC, gassed perfluorodecalins plus Coro and β-CDs; PDFdegas + CC, degassed perfluorodecalins and Coro + β-CDs; Hex, Hexenol; Hex + CC, Hexenol and Coro + β-CDs. Data are the mean of three independent replicates ± SD.

To study the relationship between gene expression and the pattern of taxane production, the expression of certain genes encoding enzymes involved in TX biosynthesis was analyzed by qRT-PCR. The studied genes and encoded enzymes were *t13αoh* (taxadiene 13-hydroxylase), involved in early synthetic steps; *bapt* (baccatin III-3-amino-13-phenylpropanoyltransferase) and *dbtnbt* (3′*N*-benzoyltransferase), which are involved in the last synthetic steps; and *pccl* (the β-phenylalanine CoA ligase), which activatesβ–phenylalanine for its attachment to the OH of C13 of baccatin III (**Figure 5**).

Gene expression was determined from 1 h to 2 days after elicitation, after previous observations that the highest expression and induction thereof occurs in this early period (Onrubia et al., 2010; Exposito et al., 2010; Onrubia et al., 2013b). Expression levels of each gene are relative to those at day 14 of culture in the growth medium (GM), using the standard curve method with the *Taxus tbc41* gene as the internal control (reference value = 1), as indicated in Section “Materials and Methods.”

In control cultures, the expression level of the *t13αoh* gene increased steadily until 24 h, decreasing thereafter (**Figure 5A**). The induction of *t13αoh* mRNA in CC-elicited cultures started after 4 h of treatment and the highest level was achieved at 48 h (*p* < 0.01) (1297 times the reference value) (**Figure 5A**). The highest accumulation of mRNA corresponding to the *t13αoh* gene was achieved under treatment with CC + PDFgas from 4 to 12 h of elicitation, with levels 11- and 16-fold higher than in CC-elicited and control cultures, respectively, suggesting a synergistic action between CC and PDFgas (**Figure 5A**). Under Hex alone, *t13αoh* gene expression was not observed, but in combination with CC, its level after 12 h was similar to the control cultures (**Figure 5A**).

We also analyzed the expression of the *pccl* gene, which encodes an acyl-CoA ligase able to convert *β*-phenylalanine into its respective derivative CoA ester. In control conditions its expression level increased steadily until 12 h (431.3 times the reference value), decreasing thereafter until 48 h (**Figure 5B**). While expression was not significantly enhanced (*p* > 0.05) by the addition of either PFDgas or PFDdegas, the presence of CC in the medium triggered a high induction: the peak level, achieved at 4 h, was 12 times higher than in the control (**Figure 5B**). However, the highest *pccl* gene expression was observed after 4 h of treatment with PDFgas + CC, being 20 times greater than in the control. The addition of PFDdegas + CC also induced an increased expression, although to a lesser extent. Under treatment with Hex, with or without CC, the *pccl* gene transcript accumulation was always significantly (*p* < 0.01) lower than in the control cultures (**Figure 5B**).

The expression of *bapt* and *dbtnbt* genes was lower than that of the *t13αoh* gene (**Figures 5C,D**), as observed in previous experiments with different *Taxus* spp. cell cultures (Onrubia et al., 2013b; Sabater-Jara et al., 2014). The CC treatment clearly enhanced the expression of the *bapt* gene, which peaked after 48 h (15-fold higher than in control cultures). Although the presence of PFDs in the medium did not clearly activate the expression of the *bapt* gene, the transcript accumulation increased dramatically under PFDgas + CC treatment until 12 h (2- and 14.5-fold higher than in CC-treated and control cultures, respectively), again suggesting a synergistic effect. In contrast, under Hex + CC the mRNA accumulation was less than with CC alone (**Figure 5C**).

The last gene involved in the TX biosynthesis, the *dbtnbt* gene, was poorly expressed in all the conditions studied, with the exception of the PFDgas + CC treatment, when it peaked at 4 h after elicitation (254.5 times the reference value) (**Figure 5D**). The addition of PFDdegas + CC also induced the expression of the *dbtnbt* gene, but the level reached was 4 times lower than with CC + PFDgas. Overall, the effect of elicitation with CC on this gene was not significant (*p* > 0.05) (**Figure 5D**), which is similar to the results obtained by Nims et al. (2006) in MeJa-elicited *T. cuspidata* cell cultures. The addition of Hex alone or with CC did not induce the expression of the *dbtnbt* gene.

### Effect of Elicitors on the Transcriptomic Profile of Elicited *Vitis vinifera* Cell Cultures

**FIGURE 6:**
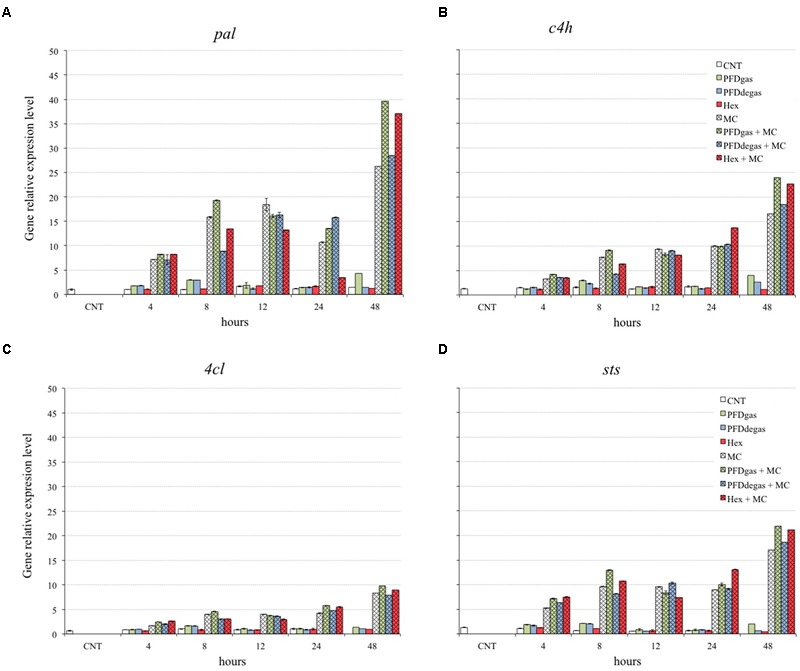
Relative expression level of genes *pal* **(A)**, *c4h* **(B)**, *4cl* **(C)**, and *sts* **(D)** in *V. vinifera* cell cultures elicited at 48 h of culture. CNT: culture medium control; PDFgas, gassed perfluorodecalins; PFDdegas, degassed perfluorodecalins; PDFgas + MC, gassed perfluorodecalins plus MeJA and β-CDs; PDFdegas + MC, degassed perfluorodecalins plus MeJA and β-CDs; Hex, Hexenol; Hex + MC, Hexenol + MeJA+ β-CDs; MC, MeJA and β-CDs. Data are the mean of three independent replicates ± SD.

To explore the relationship between gene expression and *t*-R production, the expression levels of genes encoding enzymes involved in the *t*-R biosynthesis were determined by qRT-PCR. *t*-R is biosynthesized from phenylalanine, which is transformed to cinnamic acid by the action of phenylalanine ammonia-lyase isoform 1 (PAL, an enzyme that diverts phenylalanine from its metabolic pool to the phenylpropanoid pathway). The cinnamic acid is then converted to 4-coumaroyl-CoA in two reactions catalyzed by cinnamate 4-hydroxylase (C4H, responsible for the hydroxylation of cinnamic acid) and 4-coumarate-CoA ligase isoform 1 (4CL, the enzyme that activates *trans*-coumarate). The 4-coumaroyl-CoA molecule is condensed with three malonyl-CoA units to form *t*-R by the action of stilbene synthase (STS) (Wang et al., 2015). Consequently, the expression of the *pal, c4h, 4cl* and *sts* genes was determined from 4 h to 48 h after elicitation, since the highest levels previously reported were observed in this early period (Pietrowska-Borek et al., 2014; Almagro et al., 2015; Chu et al., 2017) (**Figure 6**). For each gene, expression levels are given in relation to those obtained in the control cultures using the standard curve method with grapevine *EFα* gene as the internal control (reference value = 1), as described in Section “Materials and Methods.”

The *pal* gene was the most strongly up-regulated gene in the elicitation conditions, with maximum expression observed in the MC treatments, with or without PFDs or Hex (**Figure 6A**), which were also the conditions for the highest *t*-R production. The *pal* gene transcript levels in *V. vinifera* cell cultures treated with MC + PFDs or Hex were already 7 times higher than in control cultures after 4 h of cultivation. At 48 h of elicitation, expression levels under treatment with PFDgas + MC, PFDdegas + MC or Hex + MC were 26-, 19-, and 25-fold higher, respectively, than in control cultures, while under individual treatment with PFDgas, PFDdegas or Hex they were 9, 20, and 28-fold lower, respectively, than in combined treatments with MC (**Figure 6A**). These results demonstrate that MC acted synergistically with PFDs and Hex on the expression of the *pal* gene.

The *c4h* gene expression also increased after 4 h of elicitation (**Figure 6B**), and reached the maximum level at 48 h under treatment with MC, alone or in combination with PFDs or Hex, being up to 18 times higher than in the control cultures. At 48 h, the total transcript accumulation under elicitation with PFDgas + MC or Hex + MC was higher than the sum total when these elicitors were added separately (**Figure 6B**), suggesting a synergistic effect on the expression of the *c4h* gene.

The *4cl* gene was more poorly expressed than the other genes studied under the same experimental conditions (**Figure 6C**). Similar results were reported by Almagro et al. (2015), who observed lower RNAm accumulation of the *4cl* than *pal, c4h* or *sts* genes in MC-elicited *V. vinifera* cell cultures. The expression of the *4cl* gene was mainly increased by the addition of PFDgas + MC, especially after 48 h of elicitation (**Figure 6C**), when a significant (*p* < 0.01) increase was also observed under MC + Hex treatment (**Figure 6C**).

Expression of the *sts* gene was very low in *V. vinifera* control cultures and those treated with PFDs or Hex alone (**Figure 6D**). Maximum transcript levels were induced by PFDgas + MC after 48 h of elicitation, MC being essential for this effect (**Figure 6D**).

## Discussion

Although *T. media* cells were maintained in the PM throughout the experiment, the growth increased in control conditions, reaching the stationary phase 12 days after the beginning of the experiment. The inverse relationship between growth and total taxane production was clear in the assayed elicitation conditions, corroborating that in *Taxus* sp. cell cultures the greater the growth, the lower the production. The eliciting effect of two very different compounds, PFDs and Hex, on both growth and secondary metabolism in two different cell systems, *T. media* and *V. vinifera*, was studied. Biomass formation was enhanced by PFD treatment in *T. media*, and even more so in *V. vinifera* (**Figures 1, 2**). The addition of PFDs is thought to facilitate the supply of oxygen to the cells. *T. media* cultures tend to form clusters of 10 or more cells, while in *V. vinifera* these aggregates are smaller, which would allow a greater contact with the PFDs (**Supplementary Figure S1**). This effect of PFDgas on cell growth has already been observed in tobacco BY-2 cell cultures, which had a longer exponential growth phase when PFD represented 60% of the culture medium (Pilarek and Szewczyk, 2008). Additionally, *Petunia hybrida* protoplast cultures increased their ability to produce multicellular colonies by up to 37% when incubated for 7 days in a medium supplemented with oxygen-saturated PFD. In contrast, Syklowska-Baranek et al. (2015) observed that the addition of PFD to *T. media* hairy root cultures decreased the biomass production. It would therefore seem that each culture system may respond differently to elicitation with PFDs, and the effect probably depends on cell oxygen requirements and the resistance of roots or cell clusters to gas transfer from the culture medium.

The negative effect of some elicitors on the growth capacity of *Taxus* cell cultures is well known (Furmanowa et al., 2000; Onrubia et al., 2013b; Sabater-Jara et al., 2014). Ramirez-Estrada et al. (2015) reported that the addition of CC to another *T. media* cell line reduced the growth capacity by 14% in relation to the non-elicited control cultures. However, the addition of β-CDs alone did not inhibit the growth capacity of *Taxus* cultures (Sabater-Jara et al., 2014). MeJa also negatively affected the biomass formation of *V. vinifera* cultures and the positive effect of PFDs was not sufficiently counteractive. Interestingly, Hex was negative for cell growth in *T. media* cultures, but had no effect on this parameter in *V. vinifera* (**Figures 1, 2**). In both cell systems, the final biomass levels were lowest in the cultures treated with Hex + CC or Hex + MC, suggesting that these elicitor combinations strongly reduced cell division.

Total taxane production was highest at 24 days of culture, with a lower peak at day 12, in all the conditions studied (**Figure 3A**). The drop in total taxane yield at day 18 matches previous observations (Bonfill et al., 2003; Palazón et al., 2003; Onrubia et al., 2010, 2013b), indicating that TX and related taxanes are not end-products, but can be metabolized to other compounds with similar structures (Seki et al., 1995; Moon et al., 1998; Han et al., 2013). Conversely, the highest *t*-R levels were achieved earlier, at day 7 after elicitation (**Figure 4**).

It is striking that the main taxane found in control, Hex-, CC-, and Hex + CC-treated *Taxus* cultures was DAT (**Supplementary Table S2**). This important taxane has been extracted from *T. cuspidata* (Zhang et al., 2010) and *T. wallichiana* (McLaughlin et al., 1981), and has also been identified in cell cultures of *T. baccata* (Mroczek et al., 2000; Palazón et al., 2003; Onrubia et al., 2010) and *T. media* (Cusido et al., 2002; Exposito et al., 2010), as well as in hazelnut cell cultures (Qderi et al., 2012; Gallego et al., 2015). DAT and its 7-β-xylosyl derivative are currently considered as the best precursors for TX semi-synthesis (Rao, 1997), because they are found in higher quantities than TX in the dried bark of yew trees. 7-β-xylosyl-10-deacetyltaxol has been bioconverted to 10-deacetyltaxol with very high efficiency by a yeast culture carrying the glycoside hydrolase from *Lentinula edodes* (Liu and Zhu, 2015). Moreover, DAT is easily acetylated at the C10 position to produce TX.

The production of individual taxanes by *T. media* cells cultured in the different conditions (**Supplementary Table S2** and **Figure 3B**) indicates that the metabolic step leading to taxanes with a lateral chain attached at C13 is not rate-limiting. This may explain why the BIII and DABIII levels were not as high as in other *Taxus* cell lines, where the biosynthetic step controlled by the enzyme BAPT was flux-limiting in TX production (Nims et al., 2006; Onrubia et al., 2013b; Sabater-Jara et al., 2014). The low levels of BIII and DABIII, and the higher levels of DAT and TX obtained in this work (**Supplementary Table S2** and **Figure 3B**) suggest that the attachment of the lateral phenylisoserine chain to BIII does not reduce the production of DAT and TX. However, further studies on DAT biosynthesis are needed, since it is unclear if it is formed from the first taxane bearing this lateral chain or is a product of TX transformation.

In our study the best conditions for taxane and *t*-R production were elicitation with CC or MC, respectively. In contrast, the addition of Hex to the culture medium was detrimental for *t*-R biosynthesis, possibly because it interfered with the mechanism of action of MC, resulting in lower levels of the target compound than under MC treatment (**Figure 4**). In *T. media* cell cultures Hex also had a negative effect on TX production but enhanced the accumulation of BIII and CEPH, as discussed above (**Figure 3B**). Moreover, in *T. media* cultures, the addition of CC reversed the negative action of Hex, with TX production observed when the taxane yield was at its highest.

Our results also indicated that the MC treatment was essential for a high biotechnological production of *t*-R. In fact, the synergistic effect of the two elicitors has been previously demonstrated in *V. vinifera* (Lijavetzky et al., 2008), taxane-producing *T. baccata* (Sabater-Jara et al., 2014), and silymarin-producing *Silybum marianum* (Belchí-Navarro et al., 2011) cell cultures. The results also demonstrate that PFDs, and to a lesser extent Hex, are useful elicitors for increasing taxane and *t*-R production in *T. media* and *V. vinifera* cell cultures, respectively, when supplied together with CC or MC.

In non-elicited *T. media* cell cultures, taxanes were mainly found inside the cells, but with the addition of CC, there was a dramatic increase in their excretion to the culture medium. Similarly, *t*-R levels in the *V. vinifera* culture medium were highest with the addition of MC, with or without PFDs or Hex. This effect is mainly due to the capacity of β-CDs to form inclusion complexes with apolar compounds, such as *t*-R and taxanes, cavity (Belchí-Navarro et al., 2012; Sabater-Jara et al., 2014), besides their action as elicitors in other plant cell cultures (Zamboni et al., 2009). Therefore, β-CDs are able not only to induce the biosynthesis of metabolites such as *t-*R and taxanes, but also promote their accumulation and direct recovery. Although the mechanism of action of PFDs alone is still not understood, we observed several differences in the two cell systems studied. In *T. media*, when PFDs were added together with CC, almost half of the side-chain taxanes were found in the PFD phase, whereas no t-resveratrol was observed in this phase. It is known that *t*-resveratrol is formed in apoplasts and taxanes are synthesized within cells. This metabolic difference, together with the different chemical nature of these two compound types, could condition their different accumulation sites (culture medium or PFDs), besides the variable permeability of the two kinds of cells. These differences surely affect the biosynthesis and metabolism of *t*-R and taxanes, and consequently the contrasting response of the cell lines to elicitation.

β-CDs have been traditionally used as chelating agents to perform the bioconversion of water-insoluble precursors into the target metabolites without an organic phase in which cell viability normally decreases (Ramachandra Rao and Ravishankar, 2002). Previous studies with *Taxus* cell cultures have shown that the presence of β-CDs, alone or in combination with Coro (Ramirez-Estrada et al., 2015) or MeJa (Sabater-Jara et al., 2014), enhanced the extracellular production of total taxanes by over 90 and 80%, respectively. In addition, β-CDs reduced retro-inhibition processes and taxane toxicity for the producer cells, and prevented the enzymatic catabolism of these compounds.

When determining individual taxanes in the PFD phase, only those bearing a lateral chain were found, regardless of the experimental conditions (Data not shown). In the cultures elicited with PFDs + CC, TX levels in the PDF phase represented approximately 50% (PFDgas) and 25% (PFDdegas) of the TX yield from days 6 to 18. DAT was also found in the PDF phase from days 12 to 18, mainly when PDFs were co-administered with CC, reaching up to 50% of the DAT yield, regardless of whether the PFDs were gassed or not. Similar results were observed for CEPH, with up to 60% secreted in the PFDdegas phase in cultures co-elicited with CC. The removal of taxanes from the culture medium to the PFDdegas phase could avoid negative effects on cell growth and the production of TX and related taxanes. Consequently, PFDs could act as a sink for side chain-bearing taxanes produced in the *T. media* cell cultures, mainly when the medium was co-supplemented with CC (**Figure 3A**).

The formation of all the taxanes observed depended on the expression of the *t13αoh* gene, since all were either hydroxylated at C13 (DABIII and BIII) or underwent esterification at the hydroxylated C13 lateral chain (DAT, TX or CEPH). Although elicitation with PDFgas + CC, and to a lesser extent CC, induced the highest expression of this gene, the low expression under the other treatments was enough for inducing the activity of taxadiene 13α-hydroxylase, which is involved in the formation of the precursor of all taxanes, the polyhydroxylated taxane skeleton (**Figure 5A**).

Moreover, the expression of the *bapt* gene, which is involved in the formation of side chain-bearing taxanes such as TX, CEPH and probably DAT, was clearly enhanced by the addition of PDFs (mainly PDFgas) together with CC, and also when *T. media* cell cultures were elicited only with CC (**Figure 5C**). The positive response of this gene could explain the high TX production, especially at the end of the experiment. It is important to highlight that its transcript levels peaked soon after elicitation, whereas the highest taxane accumulation took place several days later. This could be due to the persistence of enzymatic activity long after corresponding mRNAs are no longer found in the cells (Nims et al., 2006; Katkovcinová et al., 2008; Exposito et al., 2010; Onrubia et al., 2013b). The high expression of the *bapt* gene could also lead to a high CEPH production. However, while TX production peaked at 24 days after elicitation, the highest contents of CEPH were found from days 12 to 24 (**Supplementary Table S2** and **Figure 3B**). TX and CEPH formation differs only in the last metabolic step, a bezoylation or tigloylation, respectively (Exposito et al., 2010), suggesting the enzyme that controls the tigloylation is active earlier than the DBTNBT enzyme.

The *dbtnbt* gene apparently plays a crucial role in TX formation since when Hex was added to the culture medium it was not activated and no TX was found in the cultures (**Figure 5D** and **Supplementary Table S2**). However, the Hex + CC treatment induced its expression and a corresponding increase in TX production was observed, mainly due to the action of CC (**Figures 3B, 5D**).

The PCCL enzyme is necessary for the activation of β-phenylalanine before its attachment to BIII (Ramirez-Estrada et al., 2016a). The maximum levels of the *pccl* gene were induced by CC, with or without PFDs (**Figure 5B**), which would explain the high yields of TX and CEPH produced in these conditions (**Supplementary Table S2**). However, the high content of DAT in the cultures elicited with Hex + CC could not be a consequence of the *pccl* gene, which was not expressed under this treatment.

The maximum yields of *t-*R were obtained in those cultures with the highest expression of genes involved in its biosynthesis (**Figures 4, 6**). However, although the expression levels of the four genes studied were highest in the cultures treated with PFDgas + MC, the maximum *t*-R content was reached with PFDdegas + MC (**Figure 4**). Moreover, the expression levels of these four genes were always higher in the presence of Hex + MC than MC alone (**Figure 6**), although the *t*-R production in the former did not exceed 86% of the yield under the latter conditions (**Figure 4**).

Similarly, the high extracellular *t*-R accumulation induced by β-CDs alone or in combination with MeJa was correlated with an enhanced expression of these genes (Lijavetzky et al., 2008).

In contrast with these results, a lack of a direct relationship between the expression of genes involved in the biosynthesis of taxanes and *t*-R and their production levels has been previously described in some plant cell cultures (Yang et al., 2015; Rahpeyma et al., 2017). In fact, Almagro et al. (2015) observed that the maximum expression of *sts* and *pal* genes in *V. vinifera* cell cultures took place in the presence of Coro alone, but the highest levels of *t*-R were detected with CC. Ramirez-Estrada et al. (2015) also observed that the joint action of CC increased the taxane production but the maximum expression of some genes involved in the TX biosynthetic pathway was induced by Coro alone. Therefore, other factors must also contribute to the control of a specific biosynthetic step leading to the formation of the target compounds, such as post-transcriptional and post-traductional processes, which regulate the activity of enzymes. Also, all the genes involved in the phenylpropanoid pathway, except the *sts* gene, contribute to the biosynthesis of flavonoids, lignins or other phenolic compounds.

Taken as a whole, our results show that the elicitors studied in this work induced a reprogramming of the gene expression in *Taxus* and *V. vinifera* cell cultures, which likely accounts for the differentially enhanced production of TX and related taxanes and *t*-R, respectively. Thus, whereas CC and PFDs induced the expression of the same genes to a variable extent, leading to an enhanced taxane production, Hex only improved the production of DAT. The elicitors therefore differ in their action, having synergistic or antagonistic effects.

## Conclusion

Among the elicitors assayed in this work, the joint action of PFDdegas + CC constituted the best treatment for inducing a high taxane production. Although the biomass formation was reduced by approximately 20%, the total taxane production was enhanced by 195 and 230% by CC + PFDgas and CC + PFDdegas, respectively, compared to control conditions. Similarly, in *V. vinifera* cultures the PFDdegas + MC treatment induced the highest levels of *t*-R. It can be concluded that PFDs are efficient elicitors for the production of taxanes and *t*-R in *Taxus* and *Vitis* cell cultures, although the co-presence of CC or MC is essential for this induction. The excretion of the target secondary metabolites from the producer cells either to the medium or the PDF phase in the optimum elicitation conditions undoubtedly facilitates their extraction and the downstream processes for their commercialization. The study of the expression of genes involved in the biosynthesis of these compounds has provided more information about the limiting metabolic steps for the future application of metabolic engineering techniques. However, more studies are needed, especially on enzymatic activities, in order to establish more productive cell lines. Overall, the obtained results show that to date elicited *Taxus* and *Vitis* cell cultures represent the most sustainable and eco-friendly systems for a high taxane and *t-*R production.

## Author Contributions

JP, MP, RC, and LA: conception and design of the work. HV-L, LA, EM, JP, MP, and RC: acquisition, analysis, or interpretation of data for the work. JP, MP, and RC: writing of the manuscript draft. All the authors revised and approved the final version to be published, and agreed to be accountable for all aspects of the work in ensuring that any matter regarding the accuracy or integrity of any part of the work are appropriately investigated and resolved.

## Conflict of Interest Statement

The authors declare that the research was conducted in the absence of any commercial or financial relationships that could be construed as a potential conflict of interest.

## Supplementary Material

FIGURE S1 Seven day old *T. media* and *V. vinifera* cell cultures.

TABLE S1 Sequences of the primers used to amplify genes by quantitative real-time PCR in *T. media* and *V. vinifera* cell cultures.

TABLE S2 Individual taxanes, expressed as a percentage of the total produced in *T. media* cell cultures grown for 24 days. Control; PDFgas, gassed perfluorodecalins; PFDdegas, degassed perfluorodecalins; PDFgas + CC, gassed perfluorodecalins plus Coro and β-CDs; PDFdegas + CC, degassed perfluorodecalins and Coro + β-CDs; Hex, Hexenol; Hex + CC, Hexenol and Coro + β-CDs. Data are the mean of three independent replicates ± SD.
