# Supplementary figures and images for: Perfluorodecalins and Hexenol as Inducers of Secondary Metabolism in Taxus media and Vitis vinifera Cell Cultures
Source: Front Plant Sci. 2018 Mar 16;9:335. doi: 10.3389/fpls.2018.00335 (PMC5865277; doi:10.3389/fpls.2018.00335)

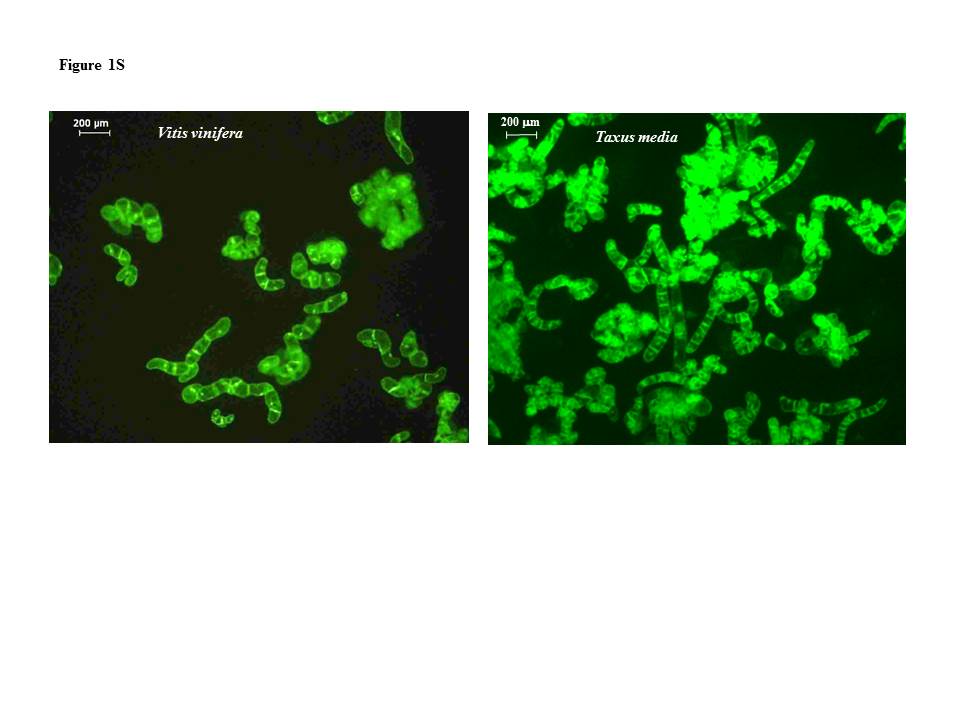

Supplement: FIGURE S1 — Seven day old T. media and V. vinifera cell cultures. [file Image_1.jpeg]
