# Supplementary material for: Perfluorodecalins and Hexenol as Inducers of Secondary Metabolism in Taxus media and Vitis vinifera Cell Cultures
Source: Front Plant Sci. 2018 Mar 16;9:335. doi: 10.3389/fpls.2018.00335 (PMC5865277; doi:10.3389/fpls.2018.00335)
Supplement: TABLE S1 — Sequences of the primers used to amplify genes by quantitative real-time PCR in T. media and V. vinifera cell cultures. [file Table_1.PDF]

Supplementary Material

Perfluorodecalins and hexenol as new inducers of secondary metabolism in *Taxus media* and *Vitis vinifera* cell cultures.

Heriberto Rafael Vidal-Limon[1], Lorena Almagro[2], Elisabeth Moyano[3], Javier Palazon[1], M. Angeles Pedreño[2], Rosa M. Cusido[1*].

*** Correspondence:** Corresponding author: rcusido@ub.edu

Table S1.

Sequences of the primers used to amplify the genes by quantitative real-time PCR in *T. media* cell cultures.

Gene	Primer sequence 5' – 3'
TBC41	Forward: CAA GAA GAA AGA GTC AGC AAA TGG
	Reverse: GGA ACG ACA TGA CAT TAT GAA TAG C
T13αOH	Forward: GCC CTT AAG CAA TTG GAA GT
	Reverse: CAG AGG AAT GGC GTT TAG AG
CoA ligase	Forward: AGC AGA CAC TAT GGA ACA
	Reverse: GCC ACA ACT CTC CTC TAT
BAPT	Forward: TAA GCA CTC TAC AAC AAC AAT GG
	Reverse: GCA TGA ACA TTA GTA TCT TGA TTC C
DBTNBT	Forward: CGG GGG GTT TGT TGT GGG ATT A
	Reverse: TTA GCC TCT CCC CTC GCC ATC T

Sequences of the primers used to amplify the genes by quantitative real-time PCR in *V. vinifera* cell cultures.

Gene	Primer sequence 5' – 3'
STS	Forward: CGAAGCAACTAGGCATGTGT
	Reverse: CTCCCCAATCCAATCCTTCA
PAL	Forward: CCGAACCGAATCAAGGACTG
	Reverse: GTTCCAGCCACTGAGACAAT
C4H	Forward: AAAGGGTGGGCAGTTCAGTT
	Reverse: GGGGGGTGAAAGGAAGATAT
4CL	Forward: CTGATGCCGCTGTTGTTTCG
	Reverse: GCAGGATTTTACCCGATGGA
EFα	Forward: GAACTGGGTGCTTGATAGGC
	Reverse: AACCAAAATATCCGGAGTAAAAGA
