# Supplementary material for: Perfluorodecalins and Hexenol as Inducers of Secondary Metabolism in Taxus media and Vitis vinifera Cell Cultures
Source: Front Plant Sci. 2018 Mar 16;9:335. doi: 10.3389/fpls.2018.00335 (PMC5865277; doi:10.3389/fpls.2018.00335)
Supplement: TABLE S2 — Individual taxanes, expressed as a percentage of the total produced in T. media cell cultures grown for 24 days. Control; PDFgas, gassed perfluorodecalins; PFDdegas, degassed perfluorodecalins; PDFgas + CC, gassed perfluorodecalins plus Coro and β-CDs; PDFdegas + CC, degassed perfluorodecalins and Coro + β-CDs; Hex, Hexenol; Hex + CC, Hexenol and Coro + β-CDs. Data are the mean of three independent replicates ± SD. [file Table_2.PDF]

Supplementary Material

Perfluorodecalins and hexenol as new inducers of secondary metabolism in *Taxus media* and *Vitis vinifera* cell cultures.

Heriberto Rafael Vidal-Limon[1], Lorena Almagro[2], Elisabeth Moyano[3], Javier Palazon[1], M. Angeles Pedreño[2], Rosa M. Cusido[1*].

*** Correspondence:** Corresponding author: rcusido@ub.edu

Table S2

Days	Treatments	DABIII	BIII	TX	DAT	CEPH
0	Control	ND	ND	ND	99.5±9.0	0.5±0.0
6	Control	0.3±0.0	9.2±0.5	3.1±0.2	84.0±7.0	3.4±0.2
	CC	ND	10.9±0.8	0.1±0.0	75.2±5.4	13.7±1.2
	PFDgas	0.4±0.0	8.9±0.5	1.9±0.1	88.5±8.0	0.3±0.0
	PFDdegas	0.5±0.0	8.4±0.4	5.4±0.3	85.1±5.3	0.6±0.0
	PFDgas+CC	ND	4.8±0.3	33.9±2.0	59.9±7.0	1.4±0.1
	PFDdegas+CC	ND	14.7±0.7	34.4±3.0	48±3.2	3±1.2
	Hex	ND	39.5±2.4	ND	55.2±3.8	5.2±0.3
	Hex+CC	ND	19.7±0.3	ND	67.7±2.9	12.6±0.9
12	Control	ND	ND	4.6±0.2	93.3±8.0	2.1±0.1
	CC	ND	5.0±0.8	8.8±1.4	59.2±3.9	27.0±1.6
	PFDgas	ND	4.8±0.3	4.1±0.2	71.2±7.0	12±0.8
	PFDdegas	22.1±2.0	1.5±0.1	5.1±0.3	69.1±7.1	2.2±0.1
	PFDgas+CC	ND	15±0.7	16.8±0.8	56.9±4	11.4±0.8
	PFDdegas+CC	ND	12.1±0.7	13.1±0.6	68.0±6.0	10.0±0.8
	Hex	ND	29.8±1.4	0.1±0.0	69.7±4.7	0.4±0.1
	Hex+CC	ND	18.9±1.6	9.6±0.7	67.2±4.9	4.3±0.2
18	Control	11±1.0	1.2±0.1	12.6±0.6	80.5±5.1	1.8±0.1
	CC	ND	6.3±0.8	3.0±0.2	60.5±4.1	30.2±1.9
	PFDgas	ND	18.9±1.0	10.2±0.5	83.8±6.1	5.6±0.3
	PFDdegas	ND	0.1±0.0	27.1±2.0	69.3±6.0	3.6±2.2
	PFDgas+CC	ND	27.5±3.0	15.6±0.8	54.6±4.1	2.3±0.1
	PFDdegas+CC	ND	23.1±2.0	16.5±0.8	55.7±6.0	4.7±0.2

	Hex	ND	12.5±0.9	0.7±0.1	86.2±6.9	0.6±0.0
	Hex+CC	ND	42.0±2.7	0.5±0.0	50.2±3.9	7.3±0.9
24	Control	6.1±0.2	0.5±0.0	6.6±0.3	83.9±7.0	2.8±0.1
	CC	ND	2.0±0.1	45.5±2.5	41.2±2.4	11.2±1.0
	PFDgas	ND	7.2±0.3	4.5±0.2	82.8±6.0	5.6±0.3
	PFDdegas	ND	8.1±0.6	5.4±0.3	81.7±6.1	4.8±0.2
	PFDgas+CC	ND	26.8±2.0	39.7±3.1	43.8±4.2	4±0.2
	PFDdegas+CC	ND	25.2±2.0	49.5±5.1	24.2±2.1	3.3±0.2
	Hex	ND	52.4±3.8	ND	45.8±3.9	1.8±0.2
	Hex+CC	0.21±0.0	16.3±0.1	35.0±1.9	44.7±3.0	3.9±0.2
